# Cu_6_Sn_5_ Whiskers Precipitated in Sn3.0Ag0.5Cu/Cu Interconnection in Concentrator Silicon Solar Cells Solder Layer

**DOI:** 10.3390/ma10040327

**Published:** 2017-03-23

**Authors:** Liang Zhang, Zhi-quan Liu, Fan Yang, Su-juan Zhong

**Affiliations:** 1 School of Mechatronic Engineering, Jiangsu Normal University, Xuzhou 221116, China; yangfan291233@126.com; 2 Institute of Metal Research, Chinese Academy of Science, Shenyang 110016, China; zqliu@imr.ac.cn; 3 State Key Laboratory of Advanced Brazing Filler Metals & Technology, Zhengzhou Research Institute of Mechanical Engineering, Zhengzhou 450001, China; zhongsj@zrime.com.cn

**Keywords:** Cu_6_Sn_5_ whiskers, Ag_3_Sn fibers, mechanical property, screw dislocation

## Abstract

Cu_6_Sn_5_ whiskers precipitated in Sn3.0Ag0.5Cu/Cu interconnection in concentrator silicon solar cells solder layer were found and investigated after reflow soldering and during aging. Ag_3_Sn fibers can be observed around Cu_6_Sn_5_ whiskers in the matrix microstructure, which can play an active effect on the reliability of interconnection. Different morphologies of Cu_6_Sn_5_ whiskers can be observed, and hexagonal rod structure is the main morphology of Cu_6_Sn_5_ whiskers. A hollow structure can be observed in hexagonal Cu_6_Sn_5_ whiskers, and a screw dislocation mechanism was used to represent the Cu_6_Sn_5_ growth. Based on mechanical property testing and finite element simulation, Cu_6_Sn_5_ whiskers were regarded as having a negative effect on the durability of Sn3.0Ag0.5Cu/Cu interconnection in concentrator silicon solar cells solder layer.

## 1. Introduction

SnPb solders—especially SnPb eutectic alloy—have been used extensively in chip attachment and surface-mount processes in the electronic packaging industry for more than five decades. Owing to the global increasing environmental and health concerns of the toxicity of Pb, international legislations (RoHS/WEEE) proposed by the EU (European Union) have banned the use of Pb in the manufacture of consumer electronic products, which has led to an extensive research and development study of lead-free solder materials [1,2,3,4]. SnAgCu solders are proposed as the one of the best alternative lead-free alloys for the traditional SnPb alloys because of their good soldering and wetting behavior on several substrate materials [5,6]. In industry, SnAgCu solders have been used as interconnected materials in different electronic devices. 

In CSP devices with capillary flow underfill, drop test results were significantly better with SnAgCu solder assembly than with SnPb eutectic alloy [7]. Comparing the induced inelastic strains in the SnAgCu and SnPb solder joints, the lead-free SnAgCu generally scored better than SnPb for QFN (Quad Flat No-lead Package) thanks to the lower creep strain rate, and for the CSP and flip chip package, SnAgCu scored worse for the more extreme loading conditions when the inelastic dissipated energy density was selected as damage parameter [8]. Kang [9] found that the Sn3.8Ag0.7Cu solders yielded three phases upon solidification: β-Sn, Ag_3_Sn, and Cu_6_Sn_5_; large plate-like pro-eutectic Ag_3_Sn structures can grow rapidly within the liquid phase, which can potentially adversely affect the mechanical behavior and reduce the fatigue life of solder joints. However, in Sn3.8Ag0.7Cu0.03Ce solder, only bulk Cu_6_Sn_5_ was found with different morphologies [10]. Moreover, the Sn3.0Ag0.5Cu solders have been proposed for use in the electronic industry, and the evolution of Ag_3_Sn and Cu_6_Sn_5_ phases should be studied further to assess the long-term reliability of SnAgCu solder joints in service.

In this work, Cu_6_Sn_5_ whiskers precipitated in Sn3.0Ag0.5Cu/Cu solder joints with deep corrosion are represented, and the growth mechanism of Cu_6_Sn_5_ whiskers was studied. The results can provide the reference for the reliability research of lead-free solder joints in service.

## 2. Experimental

**Figure 1:**
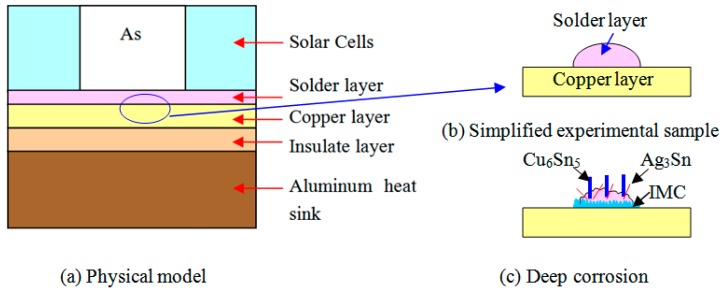
Schematic illustration of SnAgCu/Cu solder joint. (**a**) Physical; (**b**) Simplified experimental sample; (**c**) Deep corrosion.

The materials of the solder layer in solar cell are shown in Figure 1a; to simplify the research object, a simplified experimental sample (Figure 1b) was established to analyze the Sn3.0Ag0.5Cu/Cu solder joints in the concentrator silicon solar cells’ solder layer. Commercial Sn3.0Ag0.5Cu paste was put on the surface of the Cu substrate, and interconnection between Cu and SnAgCu paste was carried out by reflow soldering with peak temperature 245 °C. The samples were aged at 200 °C for 1 h. The microstructures of SnAgCu/Cu solder joint were characterized using a solution of 5% (vol.) HNO_3_ and 95% (vol.) CH_3_OH for 12 h, and ultrasonic cleaner was used to etch away the Sn matrix for 15 min; the schematic illustration is shown in Figure 1c. Then, a scanning electron microscope (Quanta200) equipped with a thermo-electron X-ray energy dispersive spectrometry (EDS) attachment was used to determine the phases in the matrix microstructure.

## 3. Results and Discussion

**Figure 2:**
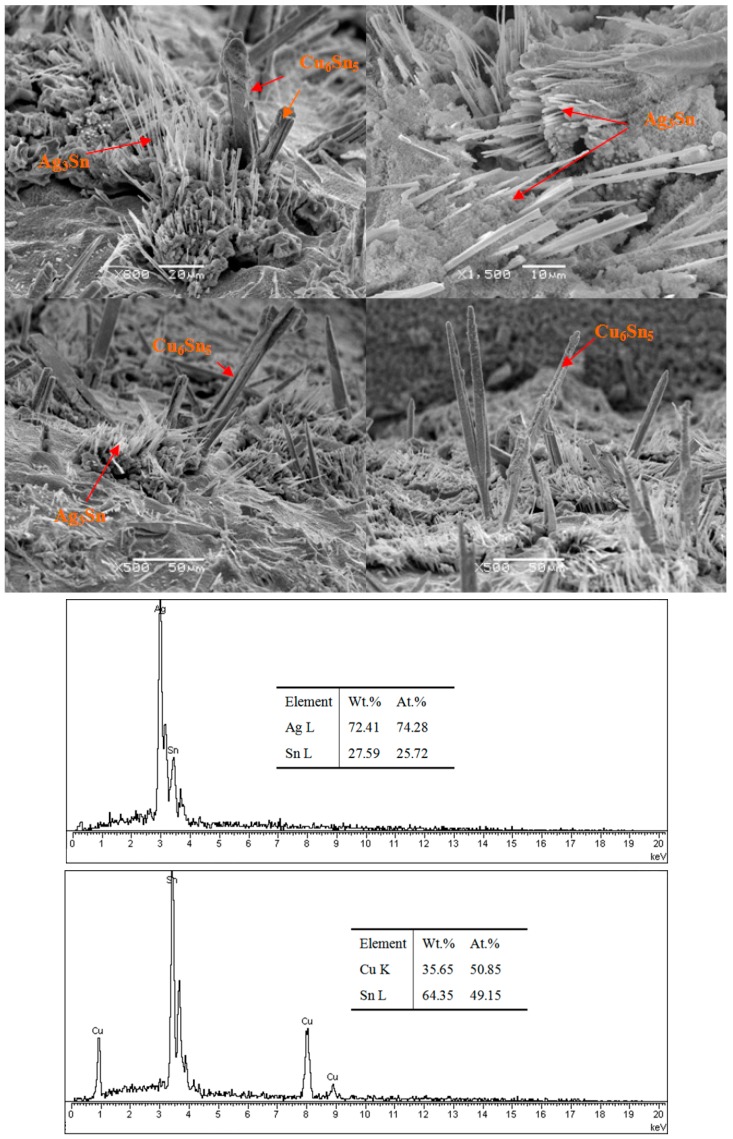
Ag_3_Sn and Cu_6_Sn_5_ phases.

Figure 2 shows the SEM images of the Sn3.0Ag0.5Cu solder joints; when the Sn matrix has been etched away, the Ag_3_Sn fibers and Cu_6_Sn_5_ whiskers can be observed. The formation of Ag_3_Sn fibers can be attributed to the solder composition bearing 3.0% Ag—not enough Ag to form large Ag_3_Sn intermetallic compound. Kim [10] found that the high Ag content alloys exhibited the formation of large Ag_3_Sn plates—especially at the solder-reaction layer interfaces—regardless of the kind of substrate. Tu [11] reported that Ag_3_Sn precipitates were plate-like in eutectic SnAg and eutectic SnAgCu, and the formation of Ag_3_Sn crystal has been demonstrated in a stress concentration region (e.g., the corner region between a solder bump and under-bump metallization). Cracks can initiate and propagate along the interface between the Ag_3_Sn and the solder. However, in this paper, for Sn3.0Ag0.5Cu solder joints after soldering, only small Ag_3_Sn fibers and no plates can be observed. Two reasons can be used to explain the formation of Ag_3_Sn fibers during aging: (1) the Ag_3_Sn particles act as pin sites and Ag atoms diffuse to nucleate and adhere to particles; (2) with the increase of thermal stress in the solder joints, small Ag_3_Sn particles can merge. With the formation of Ag_3_Sn fibers, the lengths of the fibers may be as long as tens of micrometers, and the matrix microstructure of solder joints can be strengthened. Moreover, the growth rate of Cu_6_Sn_5_ whiskers is higher than Ag_3_Sn fibers, the diameters of Cu_6_Sn_5_ whiskers are varied from 10 μm to 20 μm, and different morphologies of Cu_6_Sn_5_ whiskers can be observed—hexagonal rod structure is the main morphology of Cu_6_Sn_5_ whiskers. 

**Figure 3:**
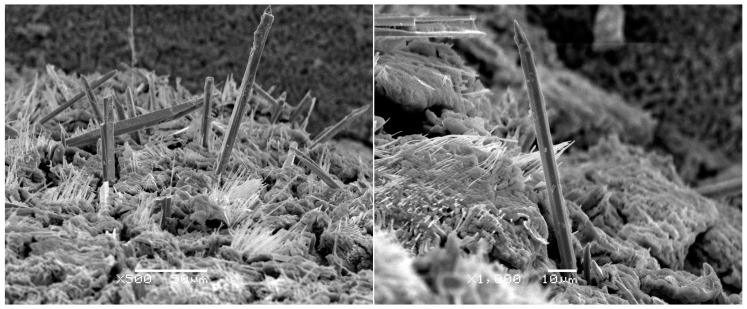
Cu_6_Sn_5_ whiskers.

**Figure 4:**
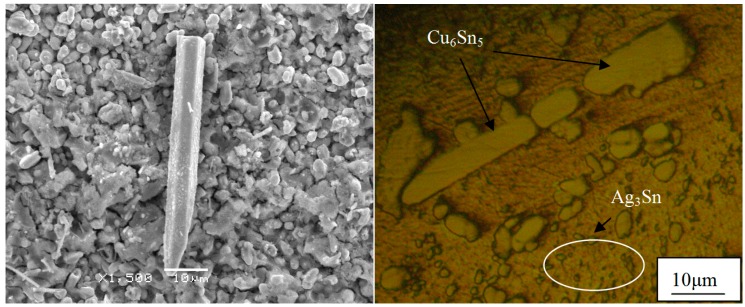
A Cu_6_Sn_5_ whisker on the surface of IMC particles.

In service, microstructure evolution is more significant for lead-free solder joints than traditional SnPb solder joints—especially for the intermetallic compounds. The reliability of solder joints is more prone to be adversely affected by intermetallic compounds; in our research, no plate Ag_3_Sn was found, Cu_6_Sn_5_ whiskers were observed, and the bulk Cu_6_Sn_5_ can lead to worse thermal fatigue resistance than joints containing Ag_3_Sn plates under specific cycling conditions [12]. For the SnAgCu/Cu solder joints, the Cu substrate can provide enough Cu atoms diffused to SnAgCu solder. In the solder matrix, the diffused Cu atoms will adhere to Cu_6_Sn_5_ particles to nucleate and react with Sn around the Cu_6_Sn_5_ particles. After several reflow soldering, Cu_6_Sn_5_ phases were also found by Tian [13], and two mechanisms of Cu_6_Sn_5_ growth were proposed: (1) the dissolution of large amounts of Cu into the solder leads to the precipitation of Cu_6_Sn_5_ in the form of long rods during solidification; and (2) the Cu_6_Sn_5_ at the interface may be broken into segments and then directly migrate into the solder joints; long Cu_6_Sn_5_ whiskers are easily broken into many small segments during in situ tensile test, and the crack can propagate and induce the failure of solder joints. The Cu_6_Sn_5_ can grow out as a hexagonal rod along a screw dislocation using the ledge mechanism [14]; this proposed mechanism of intermetallic formation incorporates the theory where whiskers are produced in metals using a single screw dislocation along the long axis of the whiskers [15]. Figure 3 shows SEM pictures of Cu_6_Sn_5_ with hexagonal rod structure; in order to represent the whole structure, the Sn matrix was etched away, and the intermetallic compound layer and Cu_6_Sn_5_ whisker can all be observed in Figure 4. The hexagonal rod structure of the Cu_6_Sn_5_ whiskers, and a cross-sectional view and small Cu_6_Sn_5_ grain in the intermetallic compound layer can be seen obviously.

**Figure 5:**
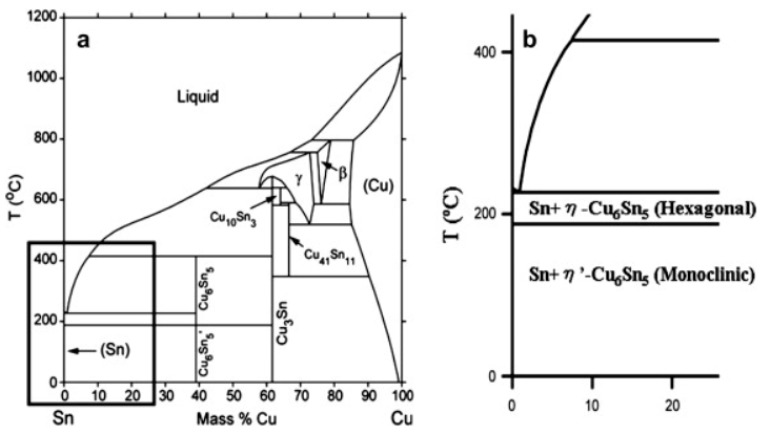
Sn–Cu phase and magnified view from the Sn-rich corner. (**a**) Cu-Sn phase diagram; (**b**) magnification of Cu-Sn phase diagram.

The Sn-Cu phase diagram [16] shown in Figure 5—which plots the Sn-0.89 Cu eutectic point and 227 °C eutectic temperature—was selected for representation in this paper to further analyze the reason for the formation of the hexagonal rod structure of the Cu_6_Sn_5_ whiskers. Two crystal structures can be observed for Cu_6_Sn_5_—monoclinic η′-Cu_6_Sn_5_ at lower temperature and hexagonal η-Cu_6_Sn_5_ at higher temperature [17], and the allotropic transformation temperature is 186 °C. In this paper, the aging temperature was 200 °C for 1 h after reflow soldering (245 °C), high enough for the phase transformation to the hexagonal rod structure of Cu_6_Sn_5_ whiskers. Moreover, the cooling time was not sufficient after high temperature aging, so the hexagonal rod structure of Cu_6_Sn_5_ did not have enough time to transform to monoclinic η′-Cu_6_Sn_5_, and thus the hexagonal rod structure is the main morphology of the Cu_6_Sn_5_ whiskers. Laurila [18] reported that the available time for the transformation into this low temperature structure was not sufficient during soldering and subsequent cooling, and so the high-temperature Cu_6_Sn_5_ remained as a metastable phase; if the temperature was near room temperature, transformation did not occur with a reasonable time because of kinetic constraints. Moreover, volume thermal expansion coefficient will increase obviously from monoclinic η′-Cu_6_Sn_5_ to hexagonal η-Cu6Sn5 [19]; therefore, the mismatch of the coefficients of thermal expansion (CTEs) of phases in the matrix will induce thermal stress in service, which will result in the failure of solder joints. 

**Figure 6:**
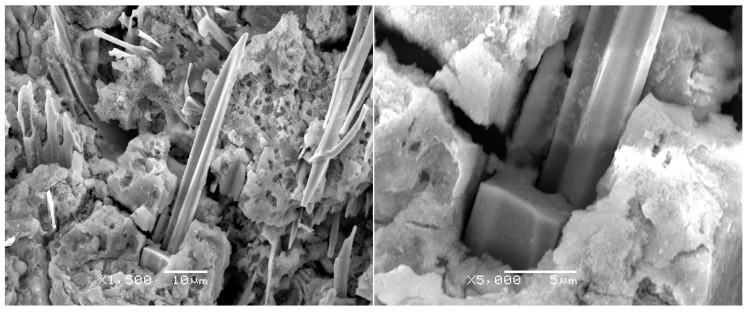
Cu_6_Sn_5_ whiskers with hole.

Figure 6 shows the Cu_6_Sn_5_ whisker, wherein the hollow in the whisker can be observed. The diameters of the hollow in the Cu_6_Sn_5_ whisker was about ~2–4 μm. As also indicated by the Cu–Sn phase diagram, the intermetallic Cu_6_Sn_5_ phase with approximately 53.5 at % Cu and 46.5 at % Sn and having the typical hollow hexagonal shape was formed in all alloys, together with the β-Sn [20]. The hollow-stick-type Cu_6_Sn_5_ forms when the core of the rod dissolves due to the higher energy of screw dislocation and lower Cu concentration, and fills with molten solder [21] during reflow soldering; after solidification, the Cu atoms will diffuse into solidified solder from the Cu substrate, and based on screw dislocation mechanism, a long hollow will appear in the Cu_6_Sn_5_ whisker. Moreover, Zhang [22] proposed that the screw dislocation core can be produced by the mismatch of atoms during the formation of Cu_6_Sn_5_, which will result in rapid lateral growth to form the special structure of the Cu_6_Sn_5_ whisker. The addition of Al into SnCu solder can significantly affect the size and morphology of Cu_6_Sn_5_ whiskers, due to epitaxial nucleation of Cu_6_Sn_5_ on either Cu_33_Al_17_ or Cu_9_A_l4_ particles [23]. Another way to control the Cu_6_Sn_5_ whisker is to prevent Cu diffusion; a nickel-based diffusion barrier is commonly used as metallization [24].

**Figure 7:**
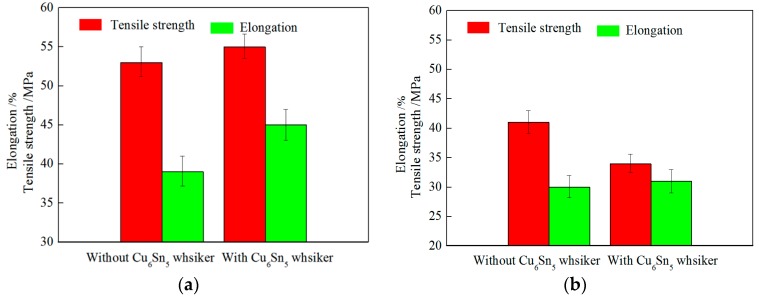
Tensile strength and elongation of solder joints with/without Cu_6_Sn_5_ whiskers. (**a**) No aging; (**b**) 150 °C aging (750 h).

**Figure 8:**
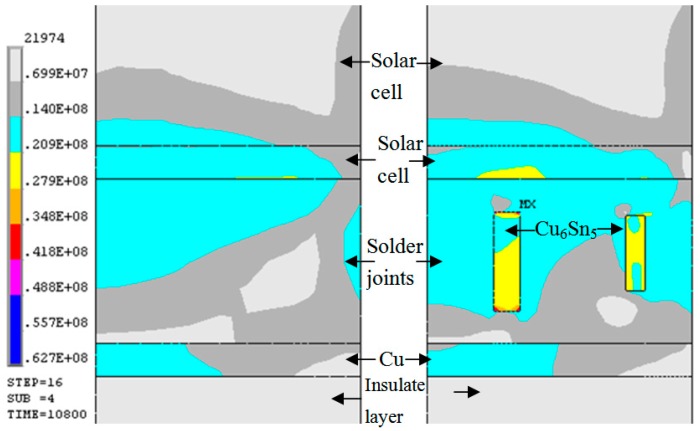
Von Mises stress in solder joints with/without Cu_6_Sn_5_ whiskers.

To analyze the mechanical properties of solder joints bearing Cu_6_Sn_5_ whiskers in the solder layer of concentrator silicon solar cells, tensile testing of solder joints with/without Cu_6_Sn_5_ whiskers was carried out (the data is shown in Figure 7). Results reveal that the tensile strength of solder joints with Cu_6_Sn_5_ whiskers was slightly higher than that without Cu_6_Sn_5_ whiskers; however, the elongation was much higher. After 150 °C aging (750 h), the tensile strength and elongation decreased significantly; the tensile strength of solder joints with Cu_6_Sn_5_ whiskers is obviously lower than that without Cu_6_Sn_5_ whiskers, which can be attributed to the degradation of properties induced by bulk brittle Cu_6_Sn_5_ phase. Finite element simulation was selected to analyze the effect of Cu_6_Sn_5_ whiskers on the properties of solder joints during aging. Figure 8 shows the von Mises stress distribution in the solder joints, and the maximum stress can be observed in the Cu_6_Sn_5_ whiskers. Therefore, these areas may be the key locations that fail most easily in service. The stress concentration can be attributed to the CTE mismatch of solar cell, Cu, solder, Cu_6_Sn_5_, and insulate layer, because the Cu_6_Sn_5_ whiskers are very hard, brittle, and noncompliant, and most stress has to be accommodated by deformation of the Cu_6_Sn_5_ whisker. So, the durability of the solder joints in the solder layer of concentrator silicon solar cells may be decreased by Cu_6_Sn_5_ whiskers in service; the inhibition of Cu_6_Sn_5_ whiskers should be studied to enhance the durability. In Sn3.8Ag0.7Cu0.03Ce solder joints [25], bulk Cu_6_Sn_5_ phase can be found in cross-section with 2D plane structure, exhibiting a wide range of sizes (~20–50 μm), and it has been demonstrated that during thermal cycling (−55 °C to 125 °C)—based on experiments and finite element simulation—the failure site was predicted to fracture near the bulk Cu_6_Sn_5_ intermetallic interface, which can be attributed to the CTE mismatch among the bulk Cu_6_Sn_5,_ Sn matrix, IMC layer, and substrates.

## 4. Conclusions

Sn3.0Ag0.5Cu/Cu solder joints in the solder layer of concentrator silicon solar cells were selected for analysis of microstructure evolution with deep corrosion. When the Sn matrix was etched away, Ag_3_Sn fibers and Cu_6_Sn_5_ whiskers could be observed. After high temperature aging, the time for cooling was not sufficient, so the hexagonal rod structure of Cu_6_Sn_5_ did not have enough time to transform to monoclinic η′-Cu_6_Sn_5_, and thus the hexagonal rod structure is the main morphology of Cu_6_Sn_5_ whiskers, and a hollow structure was found in the Cu_6_Sn_5_ whiskers. The screw dislocation mechanism was used to explain the growth of Cu_6_Sn_5_ whiskers in SnAgCu solder joints. Solder joints with Cu_6_Sn_5_ whiskers showed superior tensile strength and elongation. The degradation effect of Cu_6_Sn_5_ whiskers on mechanical properties could be demonstrated after aging for 750 h at 150 °C.
